# Injection Depot Medroxyprogesterone Acetate as an Early Postpartum Contraceptive Measure: Evaluation of Its Acceptability, Efficacy, and Impact on Lactation

**DOI:** 10.7759/cureus.66454

**Published:** 2024-08-08

**Authors:** Sonal Srivastava Garg, Bhavna Bhateja, Seema Grover, Isha Tapasvi

**Affiliations:** 1 Obstetrics and Gynecology, Guru Gobind Singh Medical College and Hospital, Faridkot, IND

**Keywords:** long-acting reversible contraceptive, lactation, postpartum contraception, depot medroxyprogesterone acetate, contraception

## Abstract

Introduction

Through its National Family Planning Programme, India has been relentlessly working to decrease society’s unmet contraception needs. The postpartum period is of paramount importance for addressing these contraceptive needs owing to alterations in fertility and coital behavior associated with childbirth.

Depot medroxyprogesterone acetate (DMPA), a long-acting reversible contraceptive, is one of the safe options available in the early postpartum period. In this study, we aimed to evaluate its efficacy and acceptability among postpartum women delivering in Guru Gobind Singh Medical College and Hospital.

Methodology

We recruited 206 early postpartum women for the study. After thorough counseling and ensuring establishment of lactation, we administered DMPA 150mg by injection intramuscularly and repeated it at intervals of three months in willing patients. We then evaluated them for their symptoms, side effects, and lactation status using a predesigned proforma either during their follow-up visits or telephonically.

Results

We found DMPA to be 100% efficacious as an early postpartum contraceptive measure. The main reasons for acceptance were its ease of use, long-term effects of a single dose, and noninterference with lactation.

However, the continuation rate for the second dose was only 18% in our study, highlighting the need for better counseling and improving awareness among our patients. Ninety-nine percent of our patients were satisfied with their lactation.

Conclusion

We found injectable DMPA used as a contraceptive in the immediate postpartum period to be a safe and effective alternate method with no deleterious effect on lactation and an acceptable side effect profile. However, more awareness programs are necessary to encourage women, especially those in low-resource areas, to continue using DMPA.

## Introduction

In India, researchers have observed the unmet need for family planning in around 13% of the married population, resulting in a considerable burden of unplanned pregnancies and subsequent abortions [1]. The overall incidence of unintended pregnancy in the first postpartum year is 12.8 per 100 women years, with 86% because of nonuse of contraception [2]. The postpartum period is therefore of paramount importance in addressing family planning needs owing to alterations in fertility and coital behavior associated with childbirth.

Over the years, India’s National Family Planning Programme has shifted its focus from merely population control to more critical issues of reducing maternal and neonatal morbidity and mortality by encouraging the use of reversible methods of contraception, thus reducing unwanted, mistimed pregnancies and avoiding high-risk pregnancies and unsafe abortions. The United Nations Population Fund has estimated that if India can fulfill the current unmet need for family planning within the next five years, it can avert 35,000 maternal deaths and 12 hundred thousand infant deaths [3].

Depot medroxyprogesterone acetate (DMPA), a long-acting reversible contraceptive, is the fourth most prevalent contraceptive worldwide and approved for use in more than 130 countries [4].

India added it to the National Family Planning Programme in 2015, and it has since proven to be one of the safe options available in the early postpartum period with a low failure rate [3]. DMPA also serves as an acceptable option for women seeking postpartum contraception but are not willing to insert a foreign body in the form of an intrauterine contraceptive device (IUCD).

DMPA acts by suppressing the mid-cycle peak of luteinizing hormone and follicle-stimulating hormone, thereby inhibiting ovulation. It also thickens the cervical mucosa and thins out the endometrial lining, thereby making implantation unfavorable [5].

However, there is much apprehension and misinformation owing to its side effects, such as alterations in the menstrual cycle, which can result in bias and discontinuation. Healthcare providers need to address this [6,7]. Previous researchers have advocated administering DMPA after six weeks postpartum considering its interference with the establishment of lactation if administered early. Theoretically, low progesterone levels in the early postpartum phase, especially in the first 72 hours, trigger an increase in prolactin, thereby stimulating the breast’s alveolar cells to secrete milk [8]. However, direct evidence from a few clinical studies demonstrates no significant negative effect of progesterone-containing contraceptives on breastfeeding [9]. Through this study, we aimed to evaluate the efficacy, continuation rate, side effect profile, and effect on lactation of DMPA when administered in the early postpartum period, that is, within one week of delivery, once lactation was established. During this time, while still admitted to the hospital, women are more compliant and receptive to contraceptive choices, and the acceptance rate is much better when compared to the acceptance rate at 6 weeks postpartum or beyond. We also aimed to allay misconceptions, increase awareness, and motivate more antenatal and postpartum patients to use injection DMPA for postpartum contraception.

Novelty of the study

Several researchers have reported on the postpartum IUCD program in Punjab, but none have conducted a study on postpartum DMPA in Punjab to determine its acceptability and continuity patterns. The results of our study helped us understand the perspectives of women in our area about this method of contraception and highlighted the need for increasing awareness and educating our women regarding this method.

Aims and objectives

Primary Objective

The primary objective of this study is to evaluate the efficacy of DMPA as a method of early postpartum contraception.

Secondary Objectives

The secondary objectives are to study the side effect profile of DMPA that women experience and its effect on lactation and to evaluate the reasons behind discontinuation of DMPA, address various misconceptions, and increase awareness.

## Materials and methods

We conducted a prospective observational study in the Department of Obstetrics and Gynaecology at Guru Gobind Singh Medical College and Hospital, Faridkot, Punjab. We recruited 206 early postpartum women from July 2022 to March 2024. We thoroughly counseled all eligible women undergoing vaginal and cesarean deliveries at the hospital regarding the basket of contraceptive options available during this period along with their benefits and side effects. We commenced contraceptive counseling during the antenatal visits in the third trimester and continued it post-delivery.

We included 206 early postpartum women in the study according to the inclusion and exclusion criteria.

Inclusion criteria

We included low-risk women aged between 18 and 35 years, weighing between 45 and 70 kg, and who gave their consent to participate in the study.

Exclusion criteria

We excluded non-consenting women and those who opted for other methods of postpartum contraception. We also excluded high-risk women such as those with morbid obesity, uncontrolled diabetes, severe preeclampsia, liver disorders, breast cancer, ischemic heart disease, or stroke.

We encouraged women to initiate breastfeeding within half an hour of vaginal delivery and two hours of cesarean section. We confirmed establishment of lactation before offering them DMPA, which we usually achieved by day 2 or 3. We conducted a thorough history-taking and physical examination and noted the details in a pre-validated proforma, along with the participants’ demographic details. We then counseled each couple regarding the dose, frequency, method of administration, side effect profile, and disturbances in menstruation and mood. We provided detailed information regarding DMPA’s reversible nature and full resumption of fertility when discontinued.

The Government of India distributes injection DMPA free of charge under the brand name Antara, in doses of 150 mg administered intramuscularly. We administered injection DMPA to consenting women under sterile conditions before discharging them any time after postpartum day 2. We confirmed adequate and satisfactory lactation before administering the injection. We documented the details regarding the date of DMPA administration, dates of follow-up visits, and subsequent doses in a card and handed it to the participants. We also advised them to visit any time in case of an emergency or any inadvertent side effect.

We evaluated women again at six weeks, two months, and three months, either through a follow-up visit or telephonically. We collected data using the questionnaire method regarding satisfaction, effect on lactation, menstrual and mood-related side effects, and any repeat pregnancy. We addressed any new apprehensions or queries related to DMPA at these visits and managed reported side effects accordingly. We also encouraged women to take subsequent DMPA doses during these follow-up visits. We followed up on those women who opted for a second dose after three months for a period of three months. The three-monthly follow-up continued for study participants opting for more doses. All women reporting amenorrhea at the follow-up underwent a urine pregnancy test to rule out pregnancy before we administered the next dose.

We collected all baseline and follow-up data in pre-validated proformas and stored them in an Excel spreadsheet. We used IBM SPSS Statistics for Windows, Version 21 (Released 2012; IBM Corp., Armonk, New York, United States) as our data analysis tool.

## Results

The majority of the women who opted for DMPA belonged to the age group of 21 to 30 years (n=154, 75%). Thirty-two women (15%) belonged to the age group of 18-20 years. Twenty women (10%) were from the age group of 31-35 years (Table 1).

**Table 1:** Socio-Demographic Profile LSCS: Lower segment cesarean section

Patient Parameters	Percentage Distribution	
Age	18-20 yrs	32 (15%)
	21-30 yrs	154 (75%)
	31-35 yrs	20 (10%)
Demographic Distribution	Urban	64 (31%)
	Rural	142 (69%)
Mode of Delivery	Vaginal	138 (67%)
	LSCS	68 (33%)
Parity	Para 1	68 (33%)
	Para 2	92 (45%)
	Para 3	36 (17%)
	Para 4	10 (5%)

As far as the demographic distribution of the population is concerned, 142 women (69%) were from rural areas, and 64 women (31%) were from urban areas (Table 1).

Sixty-seven percent (n=138) of the women who opted for DMPA had delivered vaginally and 33% (n=68) by lower segment cesarean section.

Thirty-three percent of the women were primiparas (n=68); 45% were second gravidas (n=92), 17% were third gravidas (n=36), and only 5% were fourth gravidas (n=10) because most preferred to opt for a permanent sterilization method (Table 1).

**Figure 1:**
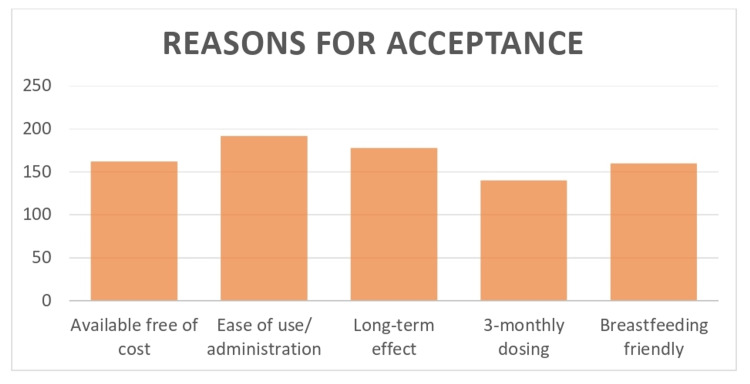
Reasons for Acceptance

Ninety-three percent of women gave its ease of use as the main reason for accepting DMPA, 86% found the long-term effect useful, 68% found the three-monthly dosing regimen convenient, 79% chose DMPA because the government provided it free of charge, and 78% considered it an advantage that DMPA had no effect on breastfeeding (Figure 1).

**Figure 2:**
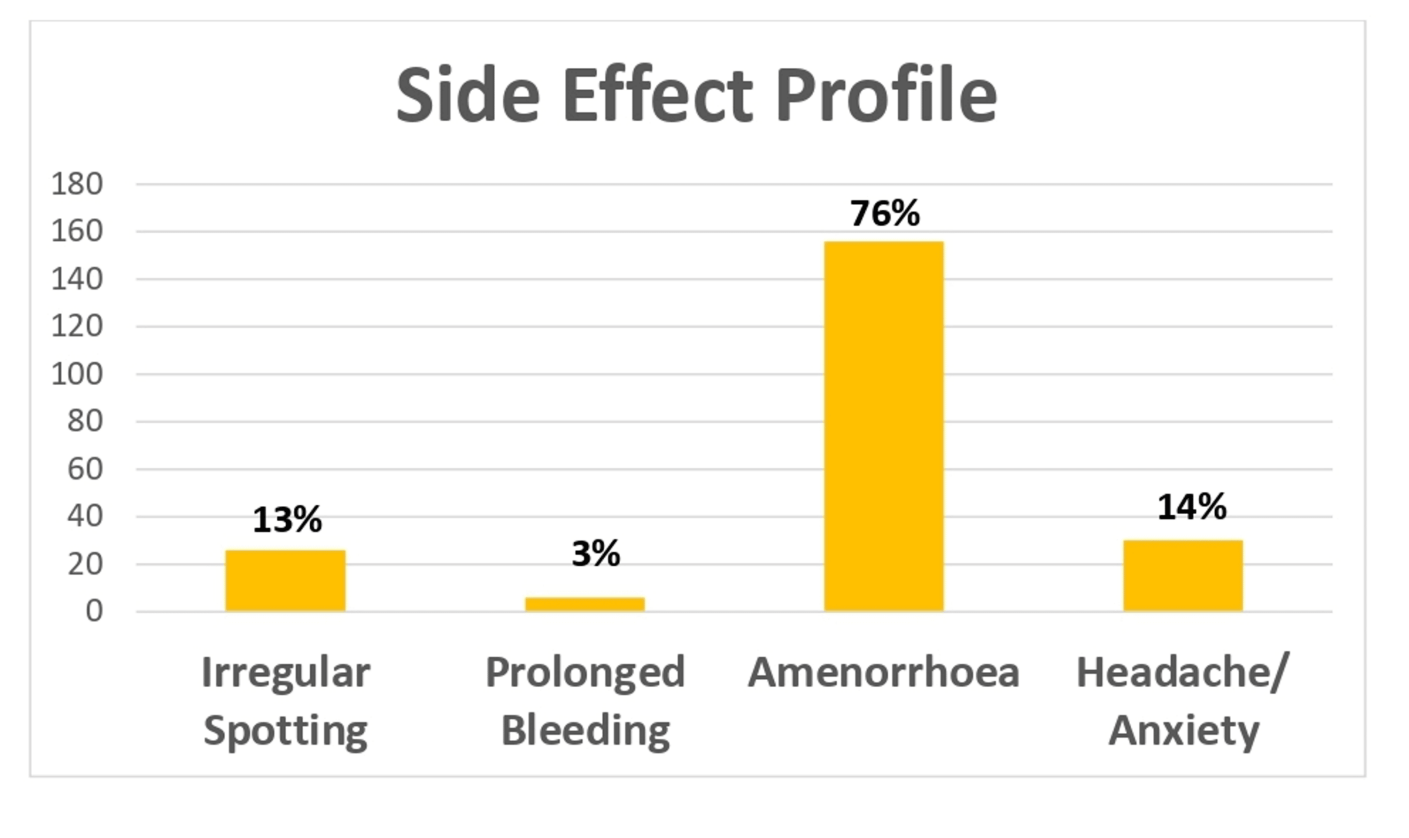
Side Effect Profile

Of all study participants, 156 (76%) reported amenorrhea as the most common side effect. Twenty-seven (13%) complained of irregular spotting, 6 (3%) had prolonged bleeding, and 29 (14%) experienced headache and anxiety (Figure 2).

Of 180 women lactating during follow-up, 178 (99%) encountered no breastfeeding problems in terms of frequency and amount.

**Figure 3:**
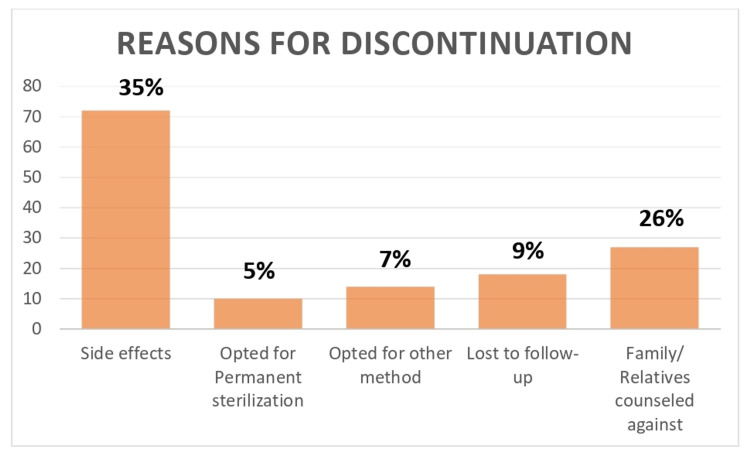
Reasons for Discontinuation

Only 38 women opted for a second dose-a continuation rate of 18% and discontinuation rate of 82% (Table 2). Of these, 27 women were from the urban population, and 11 from the rural population. Reasons for discontinuation included fear of side effects in 72 women (35%), opting for permanent sterilization in 10 women (5%), usage of some other method of contraception in 14 women (7%), and family and relatives’ advising against continuation in 27 women (26%). Eighteen women were lost to follow-up (Figure 3).

**Table 2:** Discontinuation Rate with Successive Doses

Number of DMPA dose	Total Number of Women Opting for Dose	Discontinuation Rate
1^st^	206	168 (82%)
2^nd^	38	36 (94.7%)
3^rd^	2	2 (100%)
4th	None	

Of the 38 women who continued with the second dose, 14 had spotting and 24 had amenorrhea. Twenty-two women gave side effects as the reason for discontinuation, six changed their contraception methods, and 10 cited family refusal. Only two opted for the third dose; both reported amenorrhea during their follow-up duration of three months and did not opt for the fourth dose. All women reporting amenorrhea during follow-up underwent a urine pregnancy test to confirm absence of pregnancy.

None of the participants conceived during the follow-up period. Thus, DMPA as a contraceptive method was 100% efficacious with 0% failure rate.

## Discussion

DMPA is an effective and acceptable contraceptive. Its noninterference with lactation makes it ideal postpartum. It does not interfere with sexual intercourse and has no estrogenic side effects, thus there is no increased risk of deep vein thrombosis, pulmonary embolism, stroke, or myocardial infarction. Long-term DMPA use can decrease the incidence of pelvic inflammatory disease, and it lowers the risk of endometrial cancer. Most importantly, it does not interfere with resumption of fertility upon discontinuation. Despite anecdotal clinical reports that DMPA might diminish lactation, researchers have found no direct evidence. Thus, the U.S. Medical Eligibility Criteria for Contraceptive Use lowered the WHO Medical Eligibility Criteria for Contraceptive Use risk classification for medroxyprogesterone on breastfeeding in the early postpartum period from a 3 to a 2, that is, "a condition for which the advantages of using the method generally outweigh the theoretical or proven risks’’ [9].

Most of the women in our study (75%) were in the age group of 21-30 years. These young women were generally more receptive to contraceptive counseling and usually opted for reversible contraceptives to allow future childbearing. In the study by Nigam et al., 35% were in the age group of 21-25 years [10]. According to Fonseca et al., 53.5% were in the age group of 26-30 years [11], whereas in the study by Singh et al., 61% were in the age group of 26-35 years [12].

In our study, 69% of the patients belonged to rural areas, whereas 31% were from urban areas. This disparity may be because rural women appeared more willing to get some form of contraception before discharge from the hospital and they had limited or no access to contraception once back home. In contrast, urban women have access to other methods of contraception including their partners’ use of barrier contraception. In addition, because we work in a government hospital in central Punjab, we cater more to rural patients than to urban patients. This contrasts with that reported by Nigam et al., in which 68% of the patients were from the urban population [10].

Thirty-three percent of the women in our study were primiparas and 45% were second gravidas, highlighting the need for spacing. Seventeen percent were third gravidas, and only 4% were fourth gravidas because they often opted for a permanent method of sterilization. In the study by Singh et al., only 5% of the women were primiparas, 65% were second gravidas, and 29% were grand multipara [12]. Nigam et al. and Fonseca et al. found that 54% and 78.5% of patients, respectively, had two or more children [10,11].

The most common side effect in the current study was that of amenorrhea in 75% of patients. Thirteen percent had irregular spotting, 3% had prolonged bleeding, and 14% had headache and anxiety. In the study by Singh et al. [12], 70% of the patients had amenorrhea, 50% had irregular bleeding, and 8% had headache and abdominal pain, whereas in the study by Nigam et al. [10], 59% had irregular bleeding, 6% had amenorrhea, 18% complained of weight gain, and 9% had headache and mood changes.

Ninety-three percent of the patients in our study gave ease of use as the main reason for accepting a single intramuscular DMPA injection, 86% found the long-acting effect useful, 68% found the three-monthly dosing regimen convenient, and 78% considered it an advantage that DPMA had no effect on breastfeeding. Nigam et al. also found similar reasons for acceptance in patients [10].

Our study had a high discontinuation rate of 82%, that is, only 18% of patients opted for the second dose. Reasons for discontinuation were fear of the side effects in 35%, family refusal in 26%, 7% opted for other methods, and 5% underwent permanent sterilization. Only two patients followed up for the third dose and none for the fourth. This highlights the need to counsel patients and alleviate their fears regarding the side effects. Another reason for a high discontinuation rate was that most of the patients were residents of distant rural places and referred to our hospital for delivery. Once they were back home, their families and relatives found it difficult to convince them to return for follow-up doses.

Continuation rates were a little better in other studies. In the study by Mane et al. [13], 52% of patients continued with the second dose and 46% continued with the third. In the study by Nigam et al. [10], 55% discontinued after the patient injection, 29% after the second, and 10% after the third. According to Fonseca et al. [11], the dropout rate after the first injection was 73% and 59% after the second.

Of 180 patients lactating during the follow-up period, 178 (99%) encountered no breastfeeding problems. In the study by Singhal et al. [14], also almost all the patients were satisfied with the amount and frequency of lactation. In another study, Hannon et al. [15] reported no significant difference in breastfeeding between early postpartum medroxyprogesterone recipients and nonrecipients. In fact, they observed a nonstatistically significant trend toward favorable breastfeeding outcomes in the medroxyprogesterone group.

None of the patients receiving DMPA conceived during the follow-up period, which shows 100% efficacy. Results were similar in all other studies.

The major limitation of our study was our poor continuation rate. More awareness and counseling are needed to increase acceptance of this method in our area. Also, increasing the duration and sample size of our study would have helped us to evaluate the side effect profile and acceptance rate in a better manner. Resumption of fertility after DMPA use was not assessed in our study as the follow-up period was short. 

## Conclusions

DMPA serves as an efficacious and easy method of postpartum contraception with negligible failure rates and side effects, causing no disruption in lactation, even when used in early postpartum. Clinicians may consider offering it as a first-line method for couples opting for a temporary method of sterilization, although it is necessary to spread its awareness and increase acceptance in society, more so in rural and less developed areas. In this way, we can secure women’s well-being and autonomy, thus helping us to achieve our basic aim of reducing maternal and neonatal morbidity and mortality owing to unwanted pregnancies.
